# Coding task performance in early adolescence: a large-scale controlled study into boy-girl differences

**DOI:** 10.3389/fpsyg.2013.00550

**Published:** 2013-08-27

**Authors:** Sanne Dekker, Lydia Krabbendam, Aukje Aben, Renate de Groot, Jelle Jolles

**Affiliations:** ^1^Department of Educational Neuroscience, Faculty of Psychology and Education, LEARN! Institute, VU University AmsterdamAmsterdam, Netherlands; ^2^Scientific Centre for Research in Teaching (LOOK), Ruud de Moor Centrum, Open Universiteit NederlandHeerlen, Netherlands; ^3^Centre for Learning Sciences and Technologies, Open Universiteit NederlandHeerlen, Netherlands; ^4^Faculty of Health, Medicine and Life Sciences, School for Mental Health and Neuroscience, Maastricht UniversityMaastricht, Netherlands

**Keywords:** information processing efficiency, processing speed, development, sex differences, adolescence

## Abstract

This study examined differences between boys and girls regarding efficiency of information processing in early adolescence. Three hundred and six healthy adolescents (50.3% boys) in grade 7 and 9 (aged 13 and 15, respectively) performed a coding task based on over-learned symbols. An age effect was revealed as subjects in grade 9 performed better than subjects in grade 7. Main effects for sex were found in the advantage of girls. The 25% best-performing students comprised twice as many girls as boys. The opposite pattern was found for the worst performing 25%. In addition, a main effect was found for educational track in favor of the highest track. No interaction effects were found. School grades did not explain additional variance in LDST performance. This indicates that cognitive performance is relatively independent from school performance. Student characteristics like age, sex, and education level were more important for efficiency of information processing than school performance. The findings imply that after age 13, efficiency of information processing is still developing and that girls outperform boys in this respect. The findings provide new information on the mechanisms underlying boy-girl differences in scholastic performance.

## Introduction

During an average school day, students are confronted with a wealth of information. New stimuli have to be attended to and irrelevant information has to be discarded. In a next stage of information processing, relevant information has to be analyzed and linked to previously stored material. As a result of that comparison, information can be discarded or stored in memory for later use. A core device in this process of information flow is working memory, a temporary holding device between new stimuli and already processed information (Baddeley, 1992). Students need to retrieve earlier consolidated information and search among many sources for the most relevant information. A quick and efficient accomplishment of this searching process will be beneficial to their achievement. The present paper investigates whether there are individual differences in the efficiency of information processing in early adolescence. Two narrow age groups were examined (age 13 and 15). Specifically the study sought to evaluate whether boys and girls differ with regard to speed of information processing in adolescence. Furthermore, educational track was considered as a possible source of variability, because positive relations were previously found between processing speed and intelligence (Fry and Hale, 2000; Sheppard and Vernon, 2008). Also, education effects on processing speed have been found in adults samples (Van der Elst et al., 2006; Longman et al., 2007). Given the central role of processing speed in higher cognitive functions (Salthouse, 1996; Fry and Hale, 2000), and its core importance for many aspects of performance in school, any evidence for differences in performance between boys and girls may have important implications for education.

It has been well-established that processing speed continues to improve from childhood into adolescence (age 4–17, e.g., Kail, 1991; Anderson et al., 2001; Luna et al., 2004; Nettelbeck and Burns, 2010; Coyle et al., 2011; McAuley and White, 2011). Age-related change was found to be dependent upon the processing speed measure used (Cepeda et al., 2013). Improvements in processing speed have been related to fine-tuning processes in the brain during this age period, leading to increased efficiency in brain functioning (Giedd, 2004, 2008; Gogtay et al., 2004). Following a developmental cascade model, increases in processing speed during adolescence in turn contribute to improvements in higher cognitive functions, such as working memory (Nettelbeck and Burns, 2010) and reasoning abilities (Nettelbeck and Burns, 2010; Vock et al., 2011). Also, it has been related to increases in general intelligence *g* (Coyle et al., 2011). Processing speed measures with high task demands (i.e., coding tasks) more strongly correlate with measures of higher cognitive functions than simple reaction time measures do (Nettelbeck and Burns, 2010; Cepeda et al., 2013).

Neuroimaging studies have shown that the developmental changes that take place in adolescence are different for boys and girls. There is a lag in brain development in boys compared to girls (De Bellis et al., 2001; Lenroot et al., 2007; Giedd, 2008). Total cerebral volume and gray matter volumes peak at a later age in boys (14.5 years) than in girls (10.5 years, Giedd, 2008). This suggests that next to age, sex is a possible factor influencing variability in processing speed, and that differences may be expected in young adolescents to the advantage of girls. Still, there is a lot of incongruence in research findings with respect to sex differences in processing speed during adolescence and young adulthood.

The incongruence in research findings with regard to differences between boys and girls may be related to the fact that the examined age ranges were too broad. A large study by Camarata and Woodcock (2006) showed that the magnitude of sex differences in processing speed was influenced by age. The girl advantage was relatively small in young children (9 and younger), larger in young adolescents (age 10–13), and the largest in adolescents aged 14–18. It disappeared almost completely in college students and young adults (17–34 years of age). Previous research examined age ranges that vary over a broad domain: 8–34 years (Anderson et al., 2001; Luna et al., 2004; Silveri et al., 2004; Asato et al., 2006; Camarata and Woodcock, 2006). Hence, the research by Camarata and Woodcock (2006) stresses the need to examine the issue of sex differences in processing speed during adolescence and young adulthood very specifically and in narrow age classes. Conflicting results with respect to sex differences in processing speed may also be explained by differences in processing speed measures. Generally, girls tend to outperform boys on processing speed measures that involve digits and alphabets, whereas boys are faster on simple processing speed measures (Roivainen, 2011). Furthermore, given the large effect of age and education on cognitive performance, sample sizes were often too small (<100 participants) to detect sex differences (Roivainen, 2011). Thus, sex differences in performance speed may only become prominent in large samples and in groups with narrow age ranges.

A sensitive measure to examine individual differences in processing speed is the Letter Digit Substitution Test (LDST, Jolles et al., 1995; Van der Elst et al., 2006, 2012). This coding task requires participants to match pairs of symbols according to a key. Because it involves additional cognitive processes such as working memory, visual scanning, sustained attention, response selection, interference control and monitoring, it is considered a more complex measure of processing speed (Cepeda et al., 2013). Within a given time interval (90 s), participants have to complete as many digit-letter substitutions as possible. The advantage of the LDST over other coding tasks like the Digit Symbol Substitution Test (Wechsler, 1955, 1981) or the Symbol Digit Modalities Test (Smith, 1982) relates to the use of over-learned symbols (letters and numbers) instead of abstract visual symbols. Over-learned symbols do not require complex visual processing and therefore have a lower cognitive load than abstract symbols. Hence, performance on the LDST better reflects efficiency of information processing. The LDST has already shown sensitivity to age, sex, and education level in school-aged children and older adults (Van der Elst et al., 2006, 2012).

This cross-sectional study examined three possible sources of variability in processing speed: age, sex, and educational track. Additionally, it related efficiency of information processing to real-life school performance. The study involved a total of 306 adolescents in grade 7 and grade 9 (aged 13 and 15, respectively). Participants were enrolled in one of the two highest educational tracks of Dutch secondary education. A group administration procedure was applied, enabling rapid and efficient data collection. Successful task performance was dependent on the total number of correctly completed items on the LDST. The hypotheses were: (1) adolescents in grade 9 show better task performance than adolescents in grade 7; (2) girls perform better than boys in both grades; (3) participants in the more difficult educational track perform better than participants in the less difficult track; and (4) higher school performance additionally predicted higher LDST performance. The homogenous population in terms of age range, educational track, and ethnicity, in combination with the large sample size lend the results of this study potential scientific and applied value. Finding differences between boys and girls will have implications for the fine-tuning of didactic procedures to match their working memory capacity.

## Materials and methods

### Participants

Participants were recruited from a secondary school in a town in the south-eastern part of the Netherlands. A total number of 370 students from grade 7 and grade 9 participated in the study. All students were enrolled in one of the two highest educational tracks, either *havo* or *vwo*. Every student in Dutch secondary school is enrolled in one of three educational tracks that differ in level of difficulty. Approximately 40% of all secondary school children in the Netherlands are enrolled in one of the upper two tracks (Ministry of Education, 2009). *Havo* (5 years) and *vwo* (6 years) differ in level of difficulty. A *havo* diploma gives access to professional education programs, whereas a *vwo* diploma also allows for entry into university.

LDST data were missing for two of the 370 participating students. Another 62 participants were excluded from data analysis because they met one or more of our exclusion criteria, which were: (1) repeating a class after kindergarten; (2) skipping a class after kindergarten; (3) presence of medical conditions known to influence brain development and/or cognition, such as ADHD, epilepsy, and psychosis (as indicated by a self-report questionnaire); and (4) use of medication affecting the central nervous system. This resulted in a final sample of 306 adolescents (50.3% boys), including 138 adolescents in grade 7 (*M* age = 12.9, *SD* = 0.33, range = 12.1–13.9 years, 55% boys) and 168 adolescents in grade 9 (*M* age = 15.1, *SD* = 0.36, range = 14.2–16.0 years, 46% boys). Of the 306 selected participants, 143 were enrolled at the higher general secondary educational level (46.9% boys) and 163 at pre-university educational level (53.4% boys).

### Procedure

The collaborating school agreed to fit the testing procedure into their regular school schedule. Within a week, all eligible classes of one grade had been tested. Testing took place by means of group administration and was procedurally identical for every class. Every class was tested once for a total duration of 50 min, which is equivalent to the time of one class period. Two trained investigators administered the testing protocol. One of them gave instructions to the participants and kept track of time, while the other walked around to help with potential problems. Additionally, a teacher supported task administration by keeping order in class. Testing circumstances (i.e., investigators, teacher, classroom) were kept similar for every participating class. Each testing session started by asking the participants to complete a short questionnaire on exclusion criteria. Then, several questionnaires and neuropsychological tests were administered, among which the LDST. Instructions for all tasks were given both verbally and on paper. After every instruction, participants practiced the task under supervision of the investigators. When all participants understood the task instructions, the actual task was administered. School grades were retrieved from the school's administration after study completion.

### Measures

#### Letter digit substitution test

The LDST is a coding task that is based on over-learned symbols, i.e., letters and digits (Jolles et al., 1995; Van der Elst et al., 2006). It is a measure of general information processing speed, but also involves other cognitive processes, like working memory, visual scanning, sustained attention, response selection, interference control and monitoring (Baudouin et al., 2009; Cepeda et al., 2013). At the top of the test sheet, a key is presented showing nine boxes with letters and associated numbers between 1 and 9 in a random order. Underneath the key, boxes of letters are shown with blank spaces below. Participants were instructed to replace the blank spaces with the associated digits as fast and accurately as possible, according to the key presented above. First, they practiced the procedure to ensure that they understood the purpose of the test. After completion of the practice items, participants were instructed to complete as many test items as possible within 90 s. The number of correct substitutions made in 90 s served as dependent variable. In a large sample of adults, the test-retest reliability of the LDST was high (*r* > 0.85; Van der Elst et al., 2008). Furthermore, it was shown that the LDST is sensitive to age, sex, and education level in children aged 8–15 years and in adults (Van der Elst et al., 2006, 2012).

#### School grades

We obtained grades for Dutch (native language), English (foreign language), and mathematics. Together, these grades can validly estimate school performance (Reed et al., 2010). We used the mid-semester grades, because data collection was conducted in this time period.

### Data analysis

The statistical package SPSS 20.0 was used for all data analyses. A factorial univariate Analysis of Variance (ANOVA) was conducted with number of correct substitutions made in 90 s on the LDST as dependent variable. Independent variables in the model were grade (grade 7 vs. grade 9), sex (boys vs. girls), and educational track (*havo* vs. *vwo*). Interaction effects between all pairs of independent variables were examined. Effect sizes were expressed as Cohen's *d* (Cohen, 1988), which is calculated by dividing the mean difference between each factor level by the pooled standard deviation of these factor levels. Next, LDST performance was correlated with school grades. Then, a regression analysis was performed to examine which factors predicted LDST performance. Number of correct substitutions was the dependent variable, and predictors were school year (0 = grade 7; 1 = grade 9), sex (0 = male; 1 = female), educational track (0 = havo; 1 = vwo) and the average school grade. Level of significance was α = 0.05.

## Results

Table 1 presents the number of correct substitutions on the LDST by grade, sex, and educational track. ANOVA showed significant main effects for grade [*F*_(1, 298)_ = 79.3, *p* = 0.000], for sex [*F*_(1, 298)_ = 30.4, *p* = 0.000], and for educational track [*F*_(1, 298)_ = 13.0, *p* = 0.000]. There were no interaction effects between any of the pairs of independent variables [*F*_(1, 298)_ < 1.39].

**Table 1:** **Descriptives**.

	**Boys**	**Girls**
Grade 7	46.8 (6.02)	50.2 (6.69)
Havo	46.1 (4.35)	48.9 (7.07)
Vwo	47.2 (6.91)	51.5 (6.15)
Grade 9	52.9 (7.20)	57.7 (6.97)
Havo	50.7 (7.00)	56.2 (7.04)
Vwo	54.9 (6.87)	59.4 (6.59)

Note: Mean number of correct substitutions in 90 s with standard deviations in parentheses. Havo, higher general secondary education; Vwo, pre-university education.

Results showed that adolescents in grade 9 outperformed adolescents in grade 7. The size of this effect was large (*d* = 1.09). Within both grades, a regression analysis with age, sex, and educational track showed that LDST performance was not predicted significantly by age in grade 7 [β = 0.07, *t*_(302)_ = 0.85, *p* = 0.40], nor in grade 9 [β = −0.05, *t*_(302)_ = −0.74, *p* = 0.46]. This indicates that grade 7 and grade 9 are homogeneous populations with respect to age. In both grades, girls gained significantly higher scores than boys (medium effect size, *d* = 0.63). There were no interaction effects, indicating that boys and girls did not differ significantly between grades or educational track.

**Figure 1:**
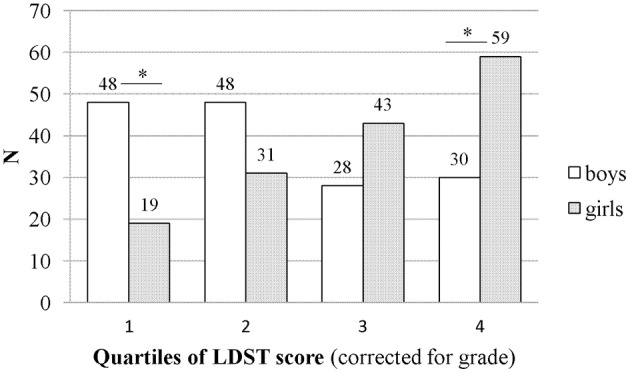
**Sex differences in LDST performance divided over quartiles.** Note: Quartile 1 = 25% lowest LDST scores; quartile 4 = 25% highest LDST scores. ^*^p < 0.001.

To take a closer look at the data and distribution of boys and girls in this sample, LDST scores were divided into quartiles from the lowest to highest scores per grade (see Figure 1). This gives more insight into the distribution of boys and girls in a group of low, medium, and good performers and enables us to draw inferences about a class situation. We found that the distribution of boys and girls differs significantly between quartiles [*X*^2^(3) = 28.8; *p* = 0.000]. It appears that students with the 25% lowest LDST scores are predominantly boys (ratio 2:1; *z* = −2.2), whereas students who belong to the 25% best performers on the LDST are predominantly girls (also ratio 2:1; *z* = 2.5). There was no significant difference in the boy:girl ratio for the second (boys: *z* = 1.3) and third (boys: *z* = −1.3) quartile.

The significant effect for educational track indicates that participants in the *vwo* track (pre-university education) scored significantly better than participants in the *havo* track (higher general secondary education). The effect size of educational track was small (*d* = 0.27).

### Relation between LDST performance and school grades

LDST score did not relate to the average school grade (*r* = 0.091, *p* = 0.112). This indicates that students who processed information more efficiently did not obtain higher grades in school than students who were slower. To examine the relation between school grades and LDST performance in more detail, school grades were also divided in quartiles. School performance of the lowest and highest performing students was related to LDST performance of students in the lowest and highest LDST quartile. No significant relations were found for any of the four contrasts (see Table 2). This indicates that real-life school performance did not relate to speed of information processing.

**Table 2:** **Correlations between school grades and LDST performance**.

**LDST quartile**	**Grade quartile**	***N***	***r***	***p***
1	1	20	0.105	0.659
1	4	11	−0.25	0.448
4	1	22	0.02	0.993
4	4	29	−0.272	0.153

The regression model (see Table 3) explained a significant proportion of variance (*R*^2^ = 0.313) in LDST score, *F*_(4, 300)_ = 34.2, *p* < 0.000. It confirmed that individual differences in LDST performance were explained by school year (β = 0.45), sex (β = 0.27) and educational track (β = 0.16), but not by school grades (β = 0.09). This showed that school grades did not explain additional variance in LDST performance next to school year, sex, and educational track.

**Table 3:** **Predictors of LDST performance**.

				**95% CI for *B***	
	***B* (SE)**	***t***	***p***	**lower**	**upper**
Intercept	38.4 (3.54)	10.8	0.000^*^	31.4	45.3
School year	7.06 (0.77)	9.22	0.000^*^	5.55	8.56
Sex	4.31 (0.76)	5.67	0.000^*^	2.81	5.80
Educational track	2.48 (0.79)	3.16	0.002^*^	0.94	4.03
Average grade	0.95 (0.51)	1.86	0.065	−0.058	1.97

Note:

* p < 0.01.

## Discussion

The purpose of this cross-sectional study was to examine whether age, sex, and educational level explained differences in the speed with which young adolescents process and manipulate information. We focused on two school years which included participants within a narrow age range of two educational tracks. Performance on the LDST was found to be dependent on grade, sex, and educational track. Results indicate that the number of correct substitutions on the LDST increased with age: adolescents in grade 9 performed significantly better than adolescents in grade 7. In both grades, girls obtained significantly higher scores than boys. The highest performing students were predominantly girls (ratio 2:1), whereas the poorest performers were predominantly boys (ratio 2:1). The rate of development was the same for boys and girls, as indicated by the lack of significant interaction effects. Furthermore, it was found that adolescents in the higher educational track outperformed adolescents in the lower educational track. School grades did not explain additional variance in LDST performance. Thus, individual differences in processing speed were explained by student characteristics, not by school performance.

The age effect found in the present study indicates that even after grade 7, there are large improvements in the efficiency with which over-learned material is processed. Thus, information processing in 15-year-old adolescents has evolved substantially by comparison to that of 13-year-old adolescents. This study therefore implies that improvements take place in the efficiency of processing of letters and digits even after childhood. Younger adolescents have less experience with these types of tasks and still have to develop automaticity in these processes. Importantly, our results furthermore indicate that boys and girls differ in processing speed and ability to manipulate new information. This suggests that the development of processing speed follows a sex-specific course in adolescence. In our sample of young adolescents, girls perform better than boys of the same age. The top 25% performers comprised twice as many girls as boys. The opposite pattern is found in the lowest scoring 25%.

Age-related increases in performance have been attributed to protracted brain development. With age, processing becomes more efficient as a result of synaptic pruning and an increase in white matter (Giedd, 2004, 2008; Gogtay et al., 2004). Protracted brain development particularly takes place in the frontal areas, to which executive control (Miller, 2005), working memory (Narayanan et al., 2005), and articulatory rehearsal (Lycke et al., 2008) have been attributed. The poorer performance of boys compared to girls is likely to be the consequence of delayed brain development in boys in the age ranges we studied (De Bellis et al., 2001; Lenroot et al., 2007). The girl advantage in LDST performance may also be related to sex differences in verbal learning, as the ability to manipulate and associate verbal material is an important determinant of successful coding task performance (Piccinin and Rabbitt, 1999; Joy et al., 2004; Cepeda et al., 2013). It has been well-established by cognitive research that girls are better verbal learners than boys (Anderson et al., 2001; Lowe et al., 2003; Meijs, 2008). Congruent with these findings, brain imaging studies have shown both structural (Schlaepfer et al., 1995; Harasty et al., 1997; Luders et al., 2005; Chen et al., 2007; Brun et al., 2009) and functional (Majeres, 1997; Baxter et al., 2003; Clements et al., 2006) differences in brain areas involved in higher order verbal functioning.

Alternatively, sex differences may be explained by motivational factors. During adolescence, large developments take place in students' beliefs and academic self-perceptions, such as their perceived competence and the value they place on doing well (see for instance Bouchey and Harter, 2005). Adolescent boys were found to have less adaptive school motivation patterns than girls, which could possibly explain their lower achievement on school-related tasks (Van Houtte, 2004; Dekker et al., 2013). Boys lower achievement may also be explained by their poorer self-regulation skills (Duckworth and Seligman, 2006). They may have suffered more than girls from the distraction that goes with assessment in a classroom setting.

The effect of educational track reported in this study indicates that higher educated adolescents process and manipulate information more quickly than lower educated adolescents. A comparable education effect has been found in adults (Van der Elst et al., 2006; Longman et al., 2007). This may be attributed to underlying differences in verbal memory. Schneider et al. (2002) found that students in higher educational tracks had better verbal memory than students in lower educational tracks, and that performance improved with age at the same rate for both educational tracks. Furthermore, given the positive relation between complex measures of processing speed and intelligence (Fry and Hale, 2000; Sheppard and Vernon, 2008), the results may also be attributed to differences in intelligence.

Yet, LDST performance was not predicted by school grades. Students who obtained higher grades, did not necessarily perform better on speeded tasks. This indicates that real-life school performance cannot be predicted by performance on one cognitive task only. Rather, numerous other factors will be involved in school grades, like motivation for learning and a structured learning environment. The findings do indicate that information processing speed as measured by the LDST may be a proxy for another dimension in (neuro)psychological functioning than school performance. More likely, it is a proxy for general intelligence. Future research should be done in this domain, to evaluate the role of processing speed in real-life school performance.

The present study does not inform about cultural differences because it was not designed to evaluate these. Previous research in (older) adults has shown that the LDST is a culturally robust test, and that performance was comparable within Europe (Houx et al., 2002) and between Europe and the USA (Moller et al., 1998). Therefore, no differences are expected in LDST performance of children of different Western countries.

In conclusion, our results support the hypothesis that the efficiency with which information is processed and manipulated in early and middle adolescence is still developing over that period. Furthermore, our findings show that sex and educational track, but not school grades, are important sources of variation for this age range. This is relevant for educational practice because many classroom activities involve processing speed. Practical implications of the research are that teachers should provide more guidance to boys, younger students, and students in lower educational tracks. They should be aware that repeated instruction may be more needed in these groups, in particular when multiple tasks are given at the same time. A higher need for clear instructions can be expected in these groups. The findings suggest that girls, older students, and students in higher educational tracks will experience less difficulty with double tasks, tasks that necessitate quick decisions, or tasks under time constraints. Future studies could usefully include older age groups to investigate the protracted development of efficiency of information processing and the stability of the sex difference over time.

### Conflict of interest statement

The authors declare that the research was conducted in the absence of any commercial or financial relationships that could be construed as a potential conflict of interest.
